# Authors’ Reply to “Additional Insights into the Analgesia Management of a High-risk Postoperative Setting”

**DOI:** 10.4274/TJAR.2026.262839

**Published:** 2026-10-30

**Authors:** Bahadır Çiftçi, Ayşe Nurmen Akın, Deniz Kızılaslan, Işılay Ayar Geçginer, Ökkeş Başak, Cem Erdoğan

**Affiliations:** 1 İstanbul Medipol University Faculty of Medicine, Department of Anaesthesiology and Reanimation, İstanbul, Türkiye; 2 Clinical Anatomy Program, Graduate School of Health Science, İstanbul Medipol University, İstanbul, Türkiye

**Keywords:** Analgesia management, high-risk patient, intensive care unit, interfascial plane blocks, M-TAPA block, pecto-intercostal fascial block

To the Editor,

We would like to thank the authors for their interest in, and constructive feedback on, our case report describing a combination of fascial plane blocks—the modified thoracoabdominal nerve block via the perichondrial approach and the pectointercostal fascial block—which we performed as part of multidisciplinary postoperative care in the intensive care unit (ICU) for a patient who underwent simultaneous liver transplant and coronary artery bypass grafting.^1^ Detailed explanations and our responses to the authors’ criticisms are provided below.

First, we address the authors’ concerns and criticisms regarding the selection and dosage of local anaesthetic (LA). A total of 80 mL of 0.125% bupivacaine was administered to our patient for the combined block, without adjuvants. As is well known, bupivacaine is already a long-acting LA; it should come as no surprise that its effect lasts for 24 hours, and examples of this situation can be found in a careful review of the literature.^2, 3, 4, 5, 6^ Since the effect of fascial plane blocks is volume-dependent, the duration of action extends as the administered volume increases.^7, 8^ When used at highly diluted concentrations, such as 0.125%, bupivacaine is known to provide effective analgesia without the absolute need for adjuvants. For instance, a recent randomized controlled study by Singh et al.^3^ demonstrated that bilateral pecto-intercostal fascial plane blocks utilizing exclusively 0.125% bupivacaine eliminated the need for rescue analgesia and provided highly effective pain control in cardiac surgery patients. Furthermore, comparative studies by Ahmed et al.^9^ and Akgul et al.^10^ evaluating bupivacaine concentrations in fascial plane blocks for major orthopedic surgeries demonstrated that 0.125% bupivacaine effectively maintained low postoperative pain scores (< 4) across a 24- to 48-hour period. While higher concentrations (such as 0.25% and 0.5%) were associated with statistically superior pain scores in these studies, the clinical difference was marginal. Importantly, Akgul et al.^10^ highlighted that higher concentrations resulted in a significantly higher incidence of motor block (e.g., 100% quadriceps weakness with 0.5% bupivacaine versus 8.7% with 0.125% bupivacaine), underscoring the clinical advantage of using a 0.125% concentration to achieve a balance between effective analgesia and minimizing motor or systemic side effects. Given that we administered a high volume (80 mL) of 0.125% bupivacaine, the outcome is consistent with the literature, which reports that this regimen provides effective, prolonged, and safe analgesia in our high-risk patient. Adjuvants are used to extend the duration of the analgesic effect of LA agents, such as bupivacaine, beyond 24 hours.

Furthermore, the total dose of bupivacaine administered at this concentration and volume (80 mL of 0.125% bupivacaine) was 100 mg in our case. For a 75-kg patient, this dose is approximately 1.33 mg kg^-1^. According to standard pharmacological guidelines, the recommended maximum dose for bupivacaine is 2-2.5 mg kg^-1 ^without epinephrine, and up to 3 mg kg^-1 ^when combined with epinephrine.^11, 12, 13^ For our 75-kg patient, the administered dose of approximately 1.33 mg kg^-1 ^is well below these established toxic thresholds. We agree that ropivacaine has a safer cardiotoxic profile than bupivacaine, provided that toxic dose limits are not exceeded. We found examples in the literature showing that bupivacaine is frequently used as a LA agent in patients undergoing cardiac surgery.^3, 4, 5, 6^ In addition, the risk of LA systemic toxicity depends not only on total dose but also on patient-related factors and systemic absorption from the injection site. We emphasize that bupivacaine, when used at a very low concentration of 0.125%, is safe even in patients undergoing high-risk cardiac surgery and provides effective analgesia for 24 hours.

Second, we agree with the authors’ emphasis on standardization and nomenclature of fascial plane blocks. The term “superficial parasternal intercostal plane block,” as agreed upon in the American Society of Regional Anesthesia and Pain Medicine and European Society of Regional Anaesthesia and Pain Therapy Delphi consensus, is important for establishing a common language and facilitating academic communication regarding fascial plane blocks.^14^ However, in daily practice, clinicians often use the terminology to which they are accustomed and reflect this usage in the literature. As De Cassai et al.^15^ also noted in the journey toward standardizing the nomenclature of interfascial plane blocks, “there is still much to do.” In their literature review, De Cassai et al.^15^ noted that for pectoral blocks, the older terms “PECS I and II” are still in use rather than the newer terms (“interpectoral” and “pecto-serratus”). If we conduct a quick and careful literature review, we can see that the term “Pecto-intercostal fascial plane block (PIFPB),” which anatomically and clearly denotes the targeted fascial plane, remains more widely used in many studies.^3-6,16-18^ Therefore, our choice of terminology is consistent with the current literature.

Finally, we agree with the comment that the Quality of Recovery (QoR) scoring system is valuable for evaluating postoperative analgesia from multiple perspectives. However, our case report describes a patient who underwent simultaneous liver transplantation and coronary artery bypass surgery, which lasted 12 hours, and was mechanically ventilated in the ICU during the postoperative period. In such a critically important and cognitively demanding scenario, these measures—ensuring the patient’s hemodynamic stability, a decrease in the numeric rating scale score, and successful extubation one hour after block administration—rather than the numerous questions required for QoR scoring, are more practical and concrete indicators of recovery and analgesic success in clinical practice.

We believe that fascial plane blocks performed under ultrasound guidance in ICUs are reliable and effective rescue analgesic methods for such high-risk patients. We would like to thank the authors once again for their valuable scientific contributions.

## References

[1] Magoon R, Choudhary N (2026). Additional insights into the analgesia management of a high-risk postoperative setting.. Turk J Anaesthesiol Reanim.

[2] Hamzi RI, Coleman SR, Pena S (2024). The effect of perineural adjuvants on superficial parasternal intercostal plane blocks in cardiac surgery: a triple-blinded randomized controlled feasibility trial.. Cureus.

[3] Singh G, Dhiraaj S, Shamshery C, Agarwal SK, Goyal P, Ambasta S (2024). To study the efficacy of ultrasound guided pecto-intercostal fascial plane block in patients undergoing midline sternotomy in open cardiac surgery: a randomized prospective comparative study.. Ann Card Anaesth.

[4] Mansour MA, Mahmoud HE, Fakhry DM, Kassim DY (2024). Comparison of the effects of transversus thoracic muscle plane block and pecto-intercostal fascial block on postoperative opioid consumption in patients undergoing open cardiac surgery: a prospective randomized study.. BMC Anesthesiol.

[5] Onur A, Onur T, Karaca Ü (2026). Evaluation of the effects of pecto-intercostal fascial plane blocks on extubation time in cardiac surgery: a retrospective study.. J Clin Med.

[6] Kaya C, Dost B, Dokmeci O, Yucel SM, Karakaya D (2022). Comparison of ultrasound-guided pecto-intercostal fascial block and transversus thoracic muscle plane block for acute poststernotomy pain management after cardiac surgery: a prospective, randomized, double-blind pilot study.. J Cardiothorac Vasc Anesth.

[7] Tulgar S, Ahıskalıoğlu A, Kilicaslan A (2025). Fascial plane blocks as the main anesthetic method: a narrative review.. Saudi J Anaesth.

[8] Sharma SK, Sonawane K, Mistry T (2026). A narrative review on fascial plane blocks - part A: anatomical foundations and mechanistic insights.. Indian J Anaesth.

[9] (2026). Ultrasound-guided suprainguinal fascia iliaca block for postoperative analgesia in patients undergoing femur fracture surgery: a randomized control trial study comparing bupivacaine (0.25%) and bupivacaine (0.125%). Annals of African medicine.

[10] Akgul IA, Canbolat N, Buget MI, Altun D, Sen C, Koltka K (2026). Comparison of local anesthetic concentration in Pericapsular Nerve Group (PENG) block for total hip arthroplasty under spinal anesthesia: a randomized controlled trial.. Pain Physician.

[11] Gropper MA, Cohen NH, Eriksson LI, Fleisher LA, Wiener-Kronish JP, Leslie K, editors. Miller’s anesthesia. 9th ed. Local anesthetics. Philadelphia: Elsevier; 2020..

[12] Song K, Blankenship RB, Dua A, Derian A. Local anesthetic toxicity. 2026 Sep 14. In: StatPearls [Internet]. Treasure Island (FL): StatPearls Publishing; 2026..

[13] Becker DE, Reed KL (2012). Local anesthetics: review of pharmacological considerations.. Anesth Prog.

[14] El-Boghdadly K, Wolmarans M, Stengel AD (2021). Standardizing nomenclature in regional anesthesia: an ASRA-ESRA Delphi consensus study of abdominal wall, paraspinal, and chest wall blocks.. Reg Anesth Pain Med.

[15] De Cassai A, Geraldini F, Tulgar S, Dost B, Munari M (2022). The journey towards nomenclature standardization for interfascial plane blocks: there is still much to do.. Minerva Anestesiol.

[16] Kaye AD, Vuong CA, Hawkins AM (2025). Pecto-intercostal fascial plane block for pain management after cardiothoracic surgery.. Curr Pain Headache Rep.

[17] Joshi P, Borde D, Apsingekar P (2024). Pecto-intercostal fascial plane block: a novel technique for analgesia in patients with sternal dehiscence.. Ann Card Anaesth.

[18] Wardhana A, Sudono H, Kurniawaty J (2025). Effectiveness of pecto-intercostal fascia plane block in reducing total postoperative opioid consumption in cardiac surgery involving sternotomy: a meta-analysis.. Ann Card Anaesth.

